# Can Pharmacological Conditioning as an Add-On Treatment Optimize Standard Pharmacological Treatment in Patients with Recent-Onset Rheumatoid Arthritis? A Proof-of-Principle Randomized Clinical Trial

**DOI:** 10.3390/ph17010110

**Published:** 2024-01-13

**Authors:** Meriem Manaï, Henriët van Middendorp, Joy A. van der Pol, Cornelia F. Allaart, Elise Dusseldorp, Dieuwke S. Veldhuijzen, Tom W. J. Huizinga, Andrea W. M. Evers

**Affiliations:** 1 Health, Medical and Neuropsychology Unit, Leiden University, P.O. Box 9500, 2300 RA Leiden, The Netherlands; meriemmanai@gmail.com (M.M.); h.vanmiddendorp@fsw.leidenuniv.nl (H.v.M.); d.s.veldhuijzen@fsw.leidenuniv.nl (D.S.V.); 2 Leiden Institute for Brain and Cognition, Leiden University, P.O. Box 9500, 2300 RA Leiden, The Netherlands; 3 The Center for Interdisciplinary Placebo Studies Leiden, P.O. Box 9500, 2300 RA Leiden, The Netherlands; 4 Department of Rheumatology, Leiden University Medical Center, P.O. Box 9600, 2300 RC Leiden, The Netherlands; j.a.van_der_pol@lumc.nl (J.A.v.d.P.); c.f.allaart@lumc.nl (C.F.A.); t.w.j.huizinga@lumc.nl (T.W.J.H.); 5 Methodology and Statistics Unit, Leiden University, P.O. Box 9500, 2300 RA Leiden, The Netherlands; elise.dusseldorp@fsw.leidenuniv.nl; 6 Department of Psychiatry, Leiden University Medical Center, P.O. Box 9600, 2300 RC Leiden, The Netherlands; 7 Medical Delta (Collaboration of Leiden University, Technical University Delft and Erasmus University), 2629 JH Delft, The Netherlands

**Keywords:** rheumatoid arthritis, pharmacological conditioning, placebo effects, clinical trial, clinical remission

## Abstract

Medication regimens using conditioning via variable reinforcement have shown similar or improved therapeutic effects as full pharmacological treatment, but evidence in patient populations is scarce. This proof-of-principle double-blind randomized clinical trial examined whether treatment effects in recent-onset rheumatoid arthritis (RA) can be optimized through pharmacological conditioning. After four months of standardized treatment (*n* = 46), patients in clinical remission (*n* = 19) were randomized to the Control group (C), continuing standardized treatment (*n* = 8), or the Pharmacological Conditioning (PC) group, receiving variable treatment according to conditioning principles (*n* = 11). After eight months, treatment was tapered and discontinued linearly (C) or variably (PC). Standard treatment led to large improvements in disease activity and HRQoL in both groups. The groups did not differ in the percentage of drug-free clinical remission obtained after conditioning or continued standard treatment. The PC group did show a larger decrease in self-reported disease activity (Cohen’s *d* = 0.9) and a smaller increase in TNF-α levels (Cohen’s *d* = 0.7) than the C group. During all phases, more differences between groups were found for the patients who followed protocol than for the intention-to-treat sample. Although the results are not conclusive, pharmacological conditioning may have some advantages in terms of disease progression and stability, especially during the conditioning phase, compared with standard clinical treatment. The effects may be particularly beneficial for patients who show a good initial response to increased medication dosages.

## 1. Introduction

Rheumatoid arthritis is a chronic inflammatory autoimmune disease characterized by painful and swollen joints, which could lead to radiological joint damage, severe disability, and premature mortality [1,2,3]. Treatment options depend on disease severity and medication tolerance. Treatments often last for a prolonged time and come with significant side effects, leading to suboptimal treatment adherence [4,5,6,7]. Standard combination therapy, consisting of methotrexate (MTX) and tapered prednisone, is successful in suppressing disease activity, improving physical functioning, preventing (progression of) joint damage, and obtaining clinical remission (with 30% of patients obtaining drug-free clinical remission) [8]. However, 25–40% of patients do not attain clinical remission on this standard combination therapy or have to discontinue therapy due to side effects or adverse events [2,9,10]. Patients who do not respond well to standard combination therapy often receive intensified and expensive pharmacological treatments, including a combination of small-molecule drugs and biologics [4,5,6,7,10]. Pharmacological conditioning [11,12,13,14,15,16] could be applied as an add-on treatment to optimize standard pharmacological treatment effects while minimizing possible side effects and health care costs and may result in more patients attaining a (drug-free) clinical remission [17].

In pharmacological conditioning, associations are formed between drug effects and drug-related contextual factors (e.g., shape, color, smell, time of day, and location). These contextual factors could then elicit drug effects similar to the active drug itself through classical conditioning mechanisms. In addition, expectations about the drug effect could lead to symptom reduction through instrumental learning mechanisms. By repeatedly pairing an active drug with an inert (placebo) treatment, this placebo treatment can thereafter evoke learned drug effects [12,13,14,15,16,18,19,20,21]. Pharmacological conditioning, therefore, has the potential to reduce or replace an active drug partly with placebo drugs, resulting in a decrease in required active drugs. Subsequently, pharmacological conditioning could provide a (partial) solution to healthcare problems such as unavailable drugs due to scarcity, harmful effects of long-term medication use, side effects, and costs.

The concept of pharmacological conditioning has only been studied in a few clinical populations for specific drugs (e.g., renal transplant, psoriasis, irritable bowel syndrome, attention-deficit hyperactivity disorder, multiple sclerosis, and Parkinson’s disease) [8,18,22,23,24,25,26]. These studies have provided insight into the optimal learning schedules to maximize pharmacological learning effects. For instance, a continuous learning schedule whereby active medication is administered on each occasion allows for learning to take place more rapidly and results in stronger learning effects than partial or intermittent learning schedules. Following continuous learning by partial or intermittent learning, whereby active medication is (partially) replaced with an inert (placebo) treatment, can extend these learned effects over a longer period of time [11,12,18,27,28,29]. 

In the current double-blind randomized clinical trial, pharmacological conditioning (PC) as an add-on treatment to standard pharmacological treatment is compared with standard pharmacological treatment alone (C) in patients diagnosed with recent-onset rheumatoid arthritis. It was expected that add-on pharmacological conditioning to standard treatment would result in a higher percentage of patients attaining a drug-free clinical remission (primary outcome) than standard treatment alone. Secondary outcomes include clinical functioning, self-reported outcomes, and laboratory outcomes [17].

## 2. Results

### 2.1. Participants

**Figure 1:**
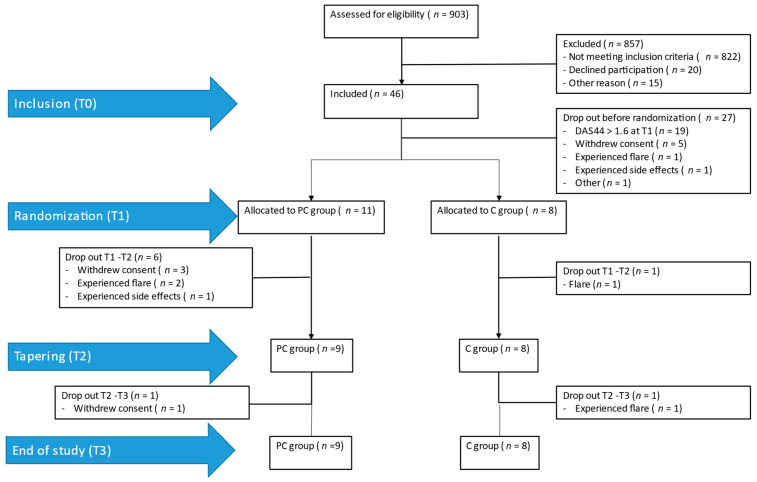
Flow diagram inclusion.

In total 46 participants were included at T0. At T1 (randomization), 27 of the 46 participants were not randomized, mainly due to a suboptimal drug response to the initial treatment, which was overall in line with the expected 50% exclusion at T1 [2]. Of these, 19 participants did not achieve clinical remission (44-joint Disease Activity Score (DAS44) > 1.6; see paragraph 4.7.1), one participant experienced a flare and discontinued before randomization; one participant withdrew due to experiencing side effects, five participants withdrew consent before randomization; and one participant received a different diagnosis and was, therefore, not randomized. Of the 19 participants that were randomized at T1 (11 to the PC group and eight to the C group), six participants discontinued protocolized treatment in the PC group (three withdrew consent of whom one participant decided to discontinue all measurements, two experienced a flare, and one experienced side effects and decided to discontinue all measurements), and one participant discontinued in the C group (experienced flare). Because two participants in the PC group decided to discontinue all measurements between T1–T2 (conditioning phase), 17 participants remained in the study at T2. Between T2–T3 (tapering phase), one participant in the PC group withdrew consent and one participant in the C group experienced a flare, leading to a total of 17 participants at T3 (nine participants in the PC group and eight participants in the C group), whereby 17 participants fall in the intention-to-treat (ITT) sample and 10 participants fall in the per-protocol (PP) sample. See Figure 1 for an overview.

Baseline characteristics of randomized participants are presented in Table 1. No significant differences between groups were found on demographic and baseline outcome measures, as indicated by a chi-square test (sex) and separate Mann–Whitney U tests. In the ITT sample, effect sizes indicated a moderate-sized larger disease activity score (DAS44) in the PC group than in the C group. In the PP sample, large-sized higher age and better physical HRQoL (RAND PCS) and moderate-sized higher DAS44, self-reported disease activity (RADAI), pain (IRGL), TNF-α, and IL-6, and lower fatigue (CIS) were found in the PC group than in the C group.

### 2.2. Primary Analysis: Percentage of Drug-Free Clinical Remission at T3

In the total sample, 41% (*n* = 7 of 17) of patients reached a drug-free clinical remission at T3 for the ITT sample, which rose to 70% (*n* = 7 of 10) in the PP sample. The percentage of patients reaching a drug-free clinical remission at T3 in the PC group (ITT: *n* = 3, 33%; PP: *n* = 3, 75%) did not differ from the percentage of drug-free clinical remission in the C group (ITT: *n* = 4, 50%; PP: *n* = 4, 67%) in both the ITT data set (*χ*^2^ (1) = 0.75, *p* = 0.387, φ = 0.21) and the PP data set (*χ*^2^ (1) = 0.08, *p* = 0.777, φ = 0.09). As can be seen from the similar raw numbers of patients achieving clinical remission in the ITT and PP samples, all patients who achieved clinical remission completely followed the protocol. 

### 2.3. Secondary Analyses

**Figure 2:**
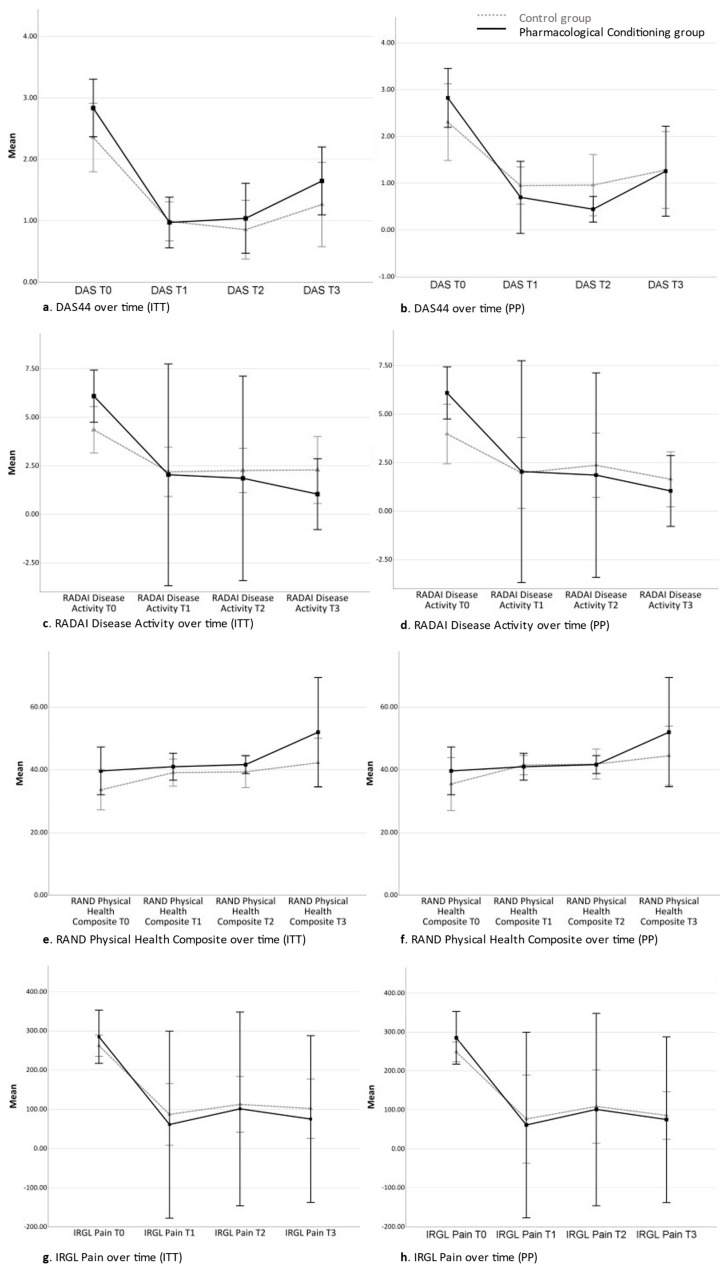

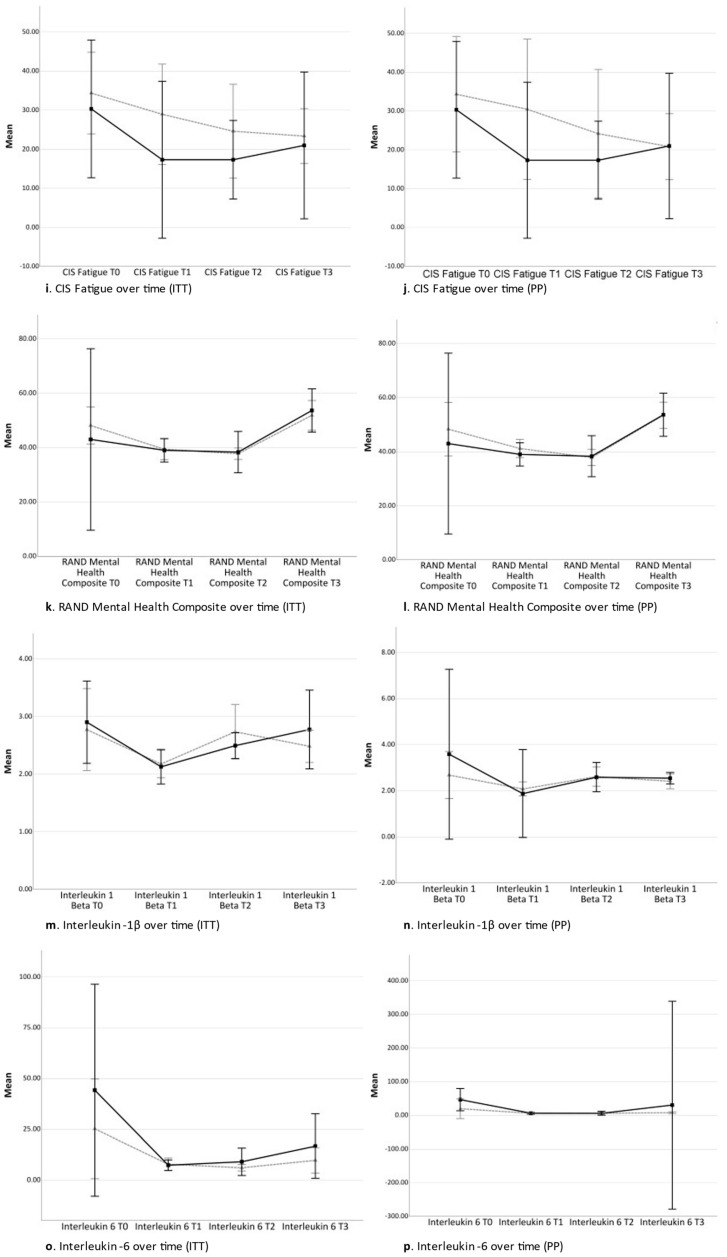

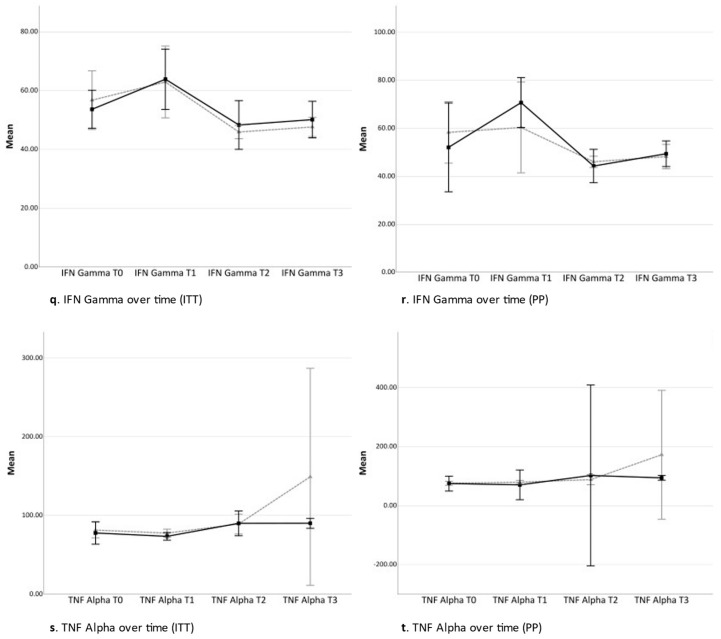
Overall time course per group from T0–T3 per outcome. (**a**) DAS44 over time (ITT). (**b**) DAS44 over time (PP). (**c**) RADAI Disease Activity over time (ITT). (**d**) RADAI Disease Activity over time (PP). (**e**) RAND Physical Health Composite over time (ITT). (**f**) RAND Physical Health Composite over time (PP). (**g**) IRGL Pain over time (ITT). (**h**) IRGL Pain over time (PP). (**i**) CIS Fatigue over time (ITT). (**j**) CIS Fatigue over time (PP). (**k**) RAND Mental Health Composite over time (ITT). (**l**) RAND Mental Health Composite over time (PP). (**m**) Interleukin-1β over time (ITT). (**n**) Interleukin-1β over time (PP). (**o**) Interleukin-6 over time (ITT). (**p**) Interleukin-6 over time (PP). (**q**) IFN Gamma over time (ITT). (**r**) IFN Gamma over time (PP). (**s**) TNF Alpha over time (ITT). (**t**) TNF Alpha over time (PP).

The overall time course per group from T0 to T3 and the specific changes between each study phase of the secondary outcome measures for the ITT and PP data can be seen in Figure 2a–t and in Table 2 (T0–T3), Table 3 (T0–T1), Table 4 (T1–T2), and Table 5 (T2–T3). Because of the large number of analyses and the small sample size, only those results showing consistent moderate-to-large-sized effect sizes across related variables (e.g., physical HRQoL measures and pro-inflammatory cytokines) or data sets (ITT vs PP) are described in the text. 

#### 2.3.1. Time Contrast between Time Point 0 and Time Point 3 (Total Study Period)

As reported in Table 2, self-reported disease activity (RADAI) showed a large-sized but non-significantly larger decrease over time for the PC group than the C group in both the ITT and the PP samples. For TNF-α, the PC group showed a moderate-sized smaller increase in TNF-α levels throughout the study period compared with the C group, which was significant in the ITT sample. All other variables did not show significant or moderate- to large-sized differences between groups. 

#### 2.3.2. Time Contrast between Time Point 0 and Time Point 1 (Acquisition Phase)

During the acquisition phase between T0 and T1, both the C group and the PC group received the same pharmacological treatment, implying that any differences between groups at this stage cannot be attributed to the pharmacological conditioning procedure. No significant differences between groups were found for this phase, but several moderate to large effect sizes were found (see Table 3). Overall, the PC group showed moderate to large-sized stronger improvements in both clinically assessed and self-reported disease activity than the C group in both the ITT and PP data sets. 

#### 2.3.3. Time Contrast between Time Point 1 and Time Point 2 (Conditioning Phase)

Table 4 shows the differences between groups for the ITT and PP data sets between T1 and T2. No significant differences between groups were found for this phase and most effect sizes indicated inconsistency across outcomes and data sets, with some showing an opposite pattern (e.g., for the cytokines) as in the previous phase.

#### 2.3.4. Time Contrast between Time Point 2 and Time Point 3 (Tapering Phase)

The differences between groups for the ITT and PP data sets between T2 and T3 are shown in Table 5. For TNF-α, a significant moderate-sized smaller increase in the PC group than in the C group was found in the ITT data set. For the other outcomes, non-significant differences were found. In the PC group, a non-significant large-sized stronger improvement in self-reported physical HRQoL was found than in the C group.

## 3. Discussion

The aim of the current proof-of-principle study was to explore for the first time the possibility of optimizing standard pharmacological treatment in patients with recent-onset rheumatoid arthritis by means of pharmacological conditioning as an add-on treatment. Using an altered clinical dosage scheme by alternating higher dosages with lower dosages, and tapering off non-linearly, we explored whether more patients would achieve drug-free clinical remission and show better physical, mental, and physiological outcomes than during standard treatment dosage schemas. The results suggest that pharmacological conditioning may have some long-term benefits, such as a sustained decrease in disease activity. Although certain study outcomes might be in favor of the conditioning group (e.g., sustained decrease in disease activity), the endpoint of pharmacological conditioning (i.e., achieving drug-free clinical remission) appears comparable to standard pharmacological treatment.

Short-term effects seem to illustrate a few advantages of the pharmacological conditioning schedule. First, while both groups showed the anticipated decrease in disease activity during the standardized treatment period, the non-conditioned group in the PP data set remained stable during the conditioning phase, whereas the conditioned group showed a further decrease. Although serum cytokine levels are very low and most of the levels are within the background fluctuations without clinical relevance, the IFN-γ levels show a larger-sized decrease in the conditioned group than the non-conditioned group during the conditioning phase, although this may have been related to the larger-sized increase in this group during the acquisition phase. Although the outcomes were all at the lowest level of the measurement scale and have to be taken with caution, pharmacological conditioning may show some beneficial effects compared with standard clinical treatment during the acquisition phase.

The results further indicate that pharmacological conditioning does not seem to have significant disadvantages compared with standard pharmacological treatment, and may even have some advantages in terms of disease progression, especially during the conditioning phase. This may indicate the potential for pharmacological conditioning for patients on long-term treatment in which there is no possibility of tapering medication.

Since during all phases, more moderate- or large-sized differences between groups were found for the patients who followed protocol than for the intention-to-treat sample, pharmacological conditioning could have beneficial effects on those patients, particularly those that show a good initial response to increased medication dosages. It would be interesting to study the characteristics of this subgroup in more detail, especially since the conditioned group also showed a higher discontinuation rate than the non-conditioned group. A possible explanation for the discrepancy between the two groups may be due to a higher intolerance rate for the higher dosages that are interspersed with lower dosages in the variable treatment schedule. These findings may suggest that only a subgroup of patients who respond well to standard pharmacological treatment and can tolerate higher dosages may profit from pharmacological conditioning. Research in genetics has also shown that specific genes involved in dopamine metabolism influence how receptive a person is to placebo effects [30,31,32]. Also, people who are generally highly optimistic and more suggestible have shown larger placebo responses than people who score lower on these characteristics [33,34]. Finally, more optimistic and suggestible people may also be more susceptible to psychological tools that can be applied in clinical practice to optimize treatment effects, such as positive framing and creating a trusting and empathetic environment between patient and health care provider [35]. The possibility to factor in genetics, personality, and the clinical environment suggests that future pharmacological conditioning interventions could be individually tailored in order to maximize treatment benefits.

### 3.1. Limitations

With regard to the feasibility of a pharmacological conditioning paradigm as an add-on to standard treatment in clinical samples, a number of issues have arisen from this proof-of-principle study that could be useful for future studies. In general, except for difficulties in the recruitment of the particular study population sought, which was mainly the result of recent changes in disease diagnostics and treatment [2,36,37], the study was generally found to be highly feasible. However, no clear conclusions of the effectiveness of pharmacological conditioning as a tool to improve chances of achieving drug-free clinical disease remission could be derived from the current study due to the relatively low sample size. Secondly, there are patients that do not respond to combined MTX and steroid therapy, or have to discontinue therapy due to side effects/adverse events [2,9,10]. Therefore, only a highly selected group of MTX and steroid users may benefit from this approach. Another point of attention is the presentation of the medication. To keep track of medication intake in case of (serious) adverse events, in the current study, medication presentation shifted from tablets distributed in a strip during the acquisition phase (standard treatment procedures) to medication in weekly containers in the conditioning and tapering phases. Patients may interpret this shift as a change to a different medication treatment, while previous treatment was effective, and respond accordingly. In addition, the concept of receiving active treatment interspersed with placebo treatment was sometimes hard to grasp and patients regularly had to be assured that the containers always contained active medication in addition to the placebo medication. Future studies could benefit from more information sessions or presenting information in various ways to ensure clarity of research goals. Moreover, potential floor effects may have occurred in both the biological as well as the clinical assays. A possible explanation may lie in the design of the study where only patients in clinical remission continued in the current study. A decline in biological and clinical outcome measures was to be expected after initial intensive treatment during the first phase of the study, with more limited room for improvement in the second phase of the study.

### 3.2. Future Perspectives

Because pharmacological conditioning may be beneficial for patients who do well on standard pharmacological treatment, future studies could investigate placebo-controlled dosage reduction in order to minimize possible side effects and socioeconomic costs and optimize the effectiveness of the pharmacological treatment. The investigation into pharmacological conditioning may also be extended to rheumatoid arthritis populations in remission on similar drugs, such as hydroxychloroquine or sulfasalazine.

For patients who need more advanced pharmacological treatments in the form of biologics, pharmacological conditioning may offer a solution to minimize the disadvantages of such treatments, including negative psychophysiological consequences of chronic drug intake, possible side effects, and costs [38,39]. Patients with severe disease activity have shown significant decreases in disease activity in response to biologicals compared with those with low disease activity, but most did not attain disease remission or low disease activity. Therefore, pharmacological conditioning could provide improvement in treatment outcomes [2]. Further, since patients with moderate disease activity compared with patients with high disease activity have a higher chance of attaining disease remission on biologics, this population may particularly benefit from placebo-controlled dosage reduction [4,6,7,40]. Because patients who do well on biologics may have already been conditioned to the positive pharmacotherapeutic effects of these types of treatments, pharmacological conditioning may provide a particularly suitable add-on intervention. In addition, as rheumatoid arthritis shares similarities in treatment options with other subpopulations within auto-immune diseases [41], pharmacological conditioning may also have potential within these populations.

## 4. Materials and Methods

A detailed overview of the study protocol was previously published [17].

### 4.1. Study Design

**Figure 3:**
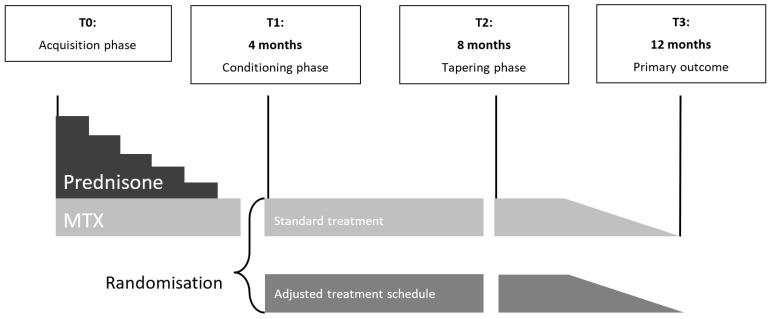
Study design over a period of 12 months with time points (T0–T3). MTX: methotrexate.

The effectiveness of a pharmacological conditioning intervention was investigated by means of a parallel-group, multicenter, randomized, double-blind, controlled superiority trial. Due to earlier signaling (i.e., before a diagnosis of rheumatoid arthritis) of symptoms and subsequent early and intensive treatment, patients were less likely to meet the classification criteria of the diagnosis of rheumatoid arthritis, and many patients were treated with MTX in an early phase of physical complaints. Because participants had to be MTX naïve to be included in the current study, only approximately 10% of ‘new’ patients with arthritis symptoms were eligible. Various steps were taken to ensure the practicality of the current study (pre-screening of patients, going multi-center, adjusting in- and exclusion criteria, and adjusting the original study design), but the inclusion rate remained too low to make a full-scale RCT feasible. Resultantly, the study was redesigned into a proof-of-principle study. Figure 3 shows the flow chart of the study design.

### 4.2. Study Population

Patients with recent-onset rheumatoid arthritis (according to the American College of Rheumatology (ACR)/European League Against Rheumatism (EULAR) 2010 classification criteria [1]) were recruited from the Department of Rheumatology at the Leiden University Medical Center (LUMC) and other hospitals in the Medical Delta vicinity in the Netherlands. Eligibility was assessed by the patient’s treating clinician according to specific inclusion and exclusion criteria [17]. An eligible patient was asked to sign informed consent after obtaining written information about the study.

### 4.3. Procedures

The study was divided into 4 periods of 4 months and was based on a previously studied clinical trial in patients with rheumatoid arthritis [2]. The current paper reports on periods 1–3 (a total of 12 months, see Figure 3 for an overview):

Period 1 (T0–T1, months 1–4): Acquisition phase. Eligible participants signed the informed consent form and started on a continuous reinforcement schedule of methotrexate (MTX) and prednisone (see intervention section).

Period 2 (T1–T2, months 5–8): Conditioning phase. Participants who did not attain clinical remission after the acquisition phase (based on the rheumatologist’s opinion, which was guided by the DAS44 (target < 1.6), were no longer eligible and were excluded from the study. In the case of clinical remission, participants were randomized without further stratification to 1 of 2 parallel groups. The Control (C) group continued with the continuous reinforcement schedule from the acquisition phase. The Pharmacological Conditioning (PC) group continued this phase on an intermittent treatment dosage of MTX, in which a high dosage of MTX was alternated with a low dosage, by interspersing active medication with placebo medication (see Section 4.4). All participants and all members of the research team who were in direct contact with the participants were blinded to the pharmacological treatment schedule that the participants received.

Period 3 (T2–T3, months 9–12): Tapering phase. Participants who were not in clinical remission discontinued with the study protocol and treatment was continued based on an individualized treatment plan. These participants were followed according to intention-to-treat (ITT): if possible and willing, participants completed further measurements. Participants who were in clinical remission were tapered off MTX, with dosages either decreasing linearly for the Control (C) group or variably for the Pharmacological Conditioning (PC) group (see Section 4.4). Participants who followed the protocol (per-protocol [PP] sample) were analyzed separately from the ITT sample.

Both groups received the same cumulative amount of MTX over all 3 periods.

### 4.4. Intervention

**Figure 4:**
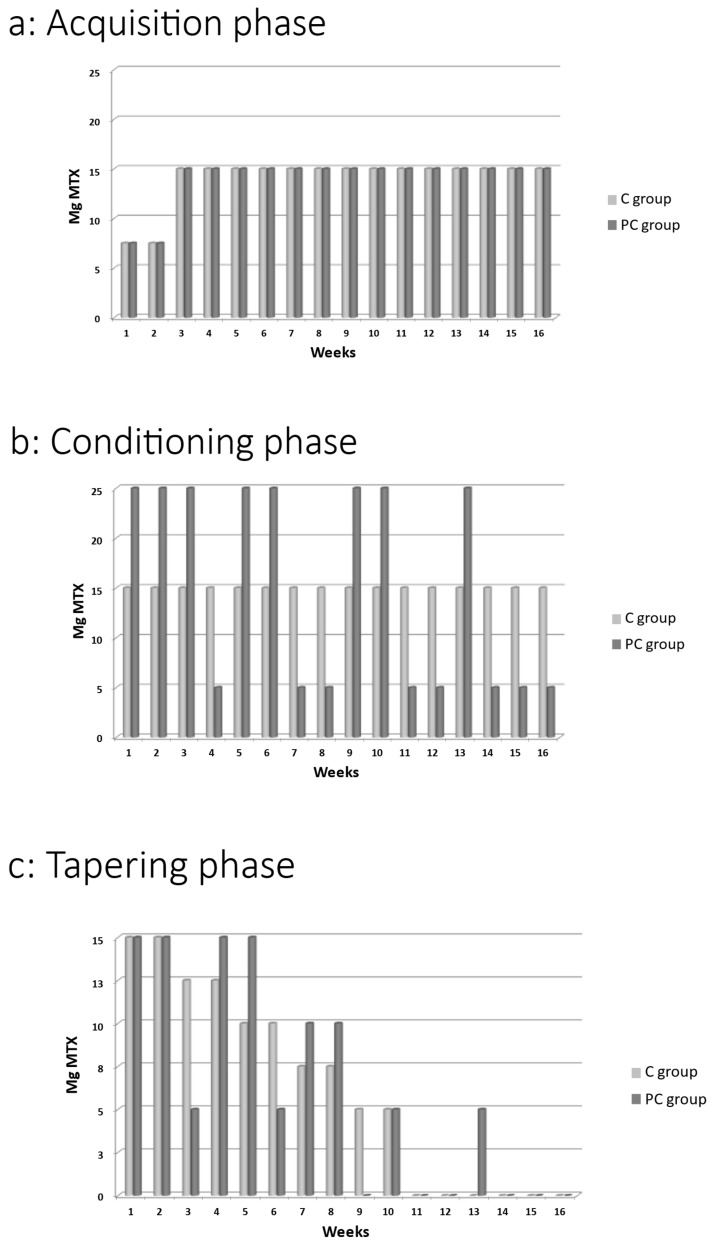
Pharmacological schedule for the separate phases. The dosage during each phase was cumulatively the same for both groups. (**a**) Pharmacological conditioning schedule for acquisition phase (T0–T1). (**b**) Pharmacological conditioning schedule for conditioning phase (T1–T2). (**c**) Pharmacological conditioning schedule for tapering phase (T2–T3). Mg: milligram; MTX: methotrexate; C group: Control group; and PC group: Pharmacological Conditioning group.

In order to allow for optimal conditioned effects, a combination of continuous reinforcement in the acquisition phase and partial reinforcement in the conditioning phase was applied [42,43,44,45,46]. During the acquisition phase (T0–T1), participants were treated with weekly dosages of 15 mg MTX, gradually increasing from a starting dose of 7.5 mg MTX. Each weekly dosage came in a bottle containing the right number of tablets. In the first 2 weeks of the acquisition phase, a total of 3 tablets of 2.5 mg MTX each (7.5 mg MTX in total) were taken each week. Starting from week 3, a total of 6 tablets of 2.5 mg MTX each (15 mg MTX in total) was taken. The medication was taken on the same day and time once a week in order to form associations between contextual factors of medication intake (conditioned stimulus: geographical location and time of day of medication intake, but also the look and feel of the medication itself) and the effect of the active medication in the body (unconditioned stimulus). See Figure 4a for an overview.

During the conditioning phase (T1–T2) and tapering phase (T2–T3), each weekly dosage came in a bottle containing 10 identical-looking tablets, whereby the ratio of active medication (2.5 mg MTX per tablet) versus placebo tablets depended on the prescribed MTX dosage for each week. During the conditioning phase, the C group received weekly bottles containing 10 tablets with 6 tablets of 2.5 mg MTX and 4 tablets of identical-looking placebos. In the PC group, a variable treatment schedule was followed. Participants in the PC group received weekly high dosages of MTX (25 mg; 10 tablets of 2.5 mg MTX), variably interspersed with weekly low dosages of MTX (5 mg: 2 tablets of 2.5 mg MTX and 8 placebo tablets). The end of the conditioning phase was marked by more frequent low dosages in the transition to the tapering phase (see Figure 4b for an overview).

During the tapering phase (T2–T3), the C group was tapered off linearly in bi-weekly decreases of 2.5 mg MTX. In the PC group, the medication was tapered off variably, whereby larger dosages were interchanged with lower dosages instead of a linear decrease in active medication dosage, whereby lower dosages were administered toward the end of this phase (see Figure 4c for an overview).

### 4.5. Assessments

Over a time period of 12 months, all participants visited the hospital every 4 months for 4 assessments in total. Assessments included a physical examination (swollen and tender joint count), laboratory evaluations (e.g., erythrocyte sedimentation rate (ESR)), and questionnaires (including but not limited to health-related quality of life). The timing of assessments aligned with usual care as much as possible.

### 4.6. Primary Outcome

The primary outcome measure was whether or not a participant achieved drug-free clinical remission based on a DAS44 value lower than 1.6 following the tapering phase (T3: 12 months after the start of treatment). Our interest was in the difference in the percentage of remitted participants between the PC and C groups.

### 4.7. Secondary Outcomes

#### 4.7.1. Disease Activity

The DAS44 is a composite disease activity index consisting of the Ritchie Articular Index (RAI [47]: ranging from 0–78), the swollen joint count among 44 joints (SJC44), the erythrocyte sedimentation rate (ESR), and general health status as assessed by the patient (GH: 0–100 visual analogue scale (VAS)). The DAS44 is computed using the following equation: DAS44 = 0.53938 × √RAI + (0.0675 × SJC44) + (0.330 × ln(ESR)) + 0.00722 × GH. DAS44 values range between 0.23 and 9.87, whereby DAS44 > 3.7 indicates high disease activity, DAS44 between 2.4 and 3.7 indicates moderate disease activity, and DAS44 between 1.6 and 2.4 indicates low disease activity. Remission is defined as DAS44 < 1.6 [48].

#### 4.7.2. Self-Reported Disease Activity

The 20-item Rheumatoid Arthritis Disease Activity Index (RADAI [49]) combines current and past (last 6 months) global disease activity, current disease activity in terms of swollen and tender joints, pain, duration of morning stiffness, and tender joints, into a single index. Higher scores indicate higher self-reported disease activity and Cronbach’s α in the current study was 0.94 at baseline.

#### 4.7.3. Physical Health-Related Quality of Life

Physical Health-Related Quality of Life (HRQoL) was assessed using the RAND Short Form-36 Health Status Inventory (RAND-SF36; [50]), the Pain subscale of the Impact of Rheumatic Diseases on General Health and Lifestyle (IRGL; [51,52]), and the Fatigue Severity subscale of the Checklist Individual Strength (CIS; [53]).

The RAND-SF36 [50] is a 36-item questionnaire that assesses 8 subscales. To measure Physical HRQoL, the Physical Health Composite Score of the RAND-SF36 was analyzed and consisted of the subscales of Physical Functioning, Role Limitations due to Physical Health Problems, Pain, and General Health Perceptions. The Hays norm-based scoring algorithm was applied, transforming raw scores into T-scores (M = 50 ± 10 in the general population) [50]. Higher scores indicate higher HRQoL and Cronbach’s α was 0.82.

The IRGL [51] Pain subscale consisting of 10 items was used to measure pain and includes assessments of the frequency of pain and swollen joints in the past month, whereby higher scores reflect higher pain symptoms. Cronbach’s α was 1.00.

The CIS is a 20-item questionnaire that assesses 4 subscales, of which the Fatigue Severity subscale was used in the analyses. This subscale consists of 8 items, whereby higher scores indicate more fatigue [53]. Cronbach’s α was 0.78.

#### 4.7.4. Mental Health-Related Quality of Life

Mental HRQoL was measured with the Mental Health Composite Score of the RAND-SF36 and consisted of the subscales of Emotional Wellbeing, Role Limitations due to Emotional Problems, Social Functioning, and Energy. Higher T-scores correspond to better mental health and Cronbach’s α was 0.60 [50].

#### 4.7.5. Cytokines

The Quantikine ELISA assay employed the quantitative sandwich enzyme immunoassay technique. A specific polyclonal antibody for human gamma interferon (IFN-y; ELISA, DIF50) and a monoclonal antibody for human interleukin (IL) 1ß (ELISA, DLB50), IL-6 (ELISA, D6050), and tumor necrosis factor alpha (TNF-α; ELISA, DTA00D) were pre-coated onto a microplate. Standards, QC (Quantikine Immunoassay Control Group 1; QC01-1 or Quantikine Immunoassay Control Group248), and samples were pipetted into the wells and any antigen present was bound by the immobilized antibody. After washing away any unbound substances, an enzyme-linked polyclonal or monoclonal antibody specific to human antigens was added to the wells. Following a wash to remove any unbound antibody-enzyme reagent, a substrate solution was added to the wells, and the color was developed in proportion to the amount of antigen bound in the initial step. The color development was stopped and the intensity of the color was measured. All ELISA and QC assays were executed according to the manufacturer’s manual (R&D Systems, Minneapolis, MN, USA).

### 4.8. Statistical Analyses

Data analyses were performed with SPSS Statistics (IBM Corporation, Armonk, NY, USA). Due to the proof-of-principle character of the current study, the focus was shifted from the statistical significance of group differences (*p* < 0.05) to a more descriptive and group-size independent effect size [54] description of group differences across time points. In the case of categorical variables, phi effect size was calculated, where a value of φ = 0.1 is considered to be a small effect, 0.3 a medium effect, and 0.5 a large effect. For continuous variables, Cohen’s *d* effect sizes were calculated, with an effect size of 0.2 being considered small, an effect size of 0.5 medium, and an effect size of 0.8 or higher being considered a large effect [55]. This study focused on effect sizes of at least moderate size in order to prevent overinterpretation of the data. Also, looking at the separate phases of the study in addition to the general effect over the entire study period provided more insight into the underlying mechanisms that perhaps are specific for each phase of the current trial. To this aim, standardized mean change scores (effect sizes) for T0–T3, T0–T1, T1–T2, and T2–T3 were calculated by dividing the difference between the mean change score between the PC group and the C group by the pooled standard deviation of the change scores. The formula of the calculation of the effect sizes (standardized mean change score differences) between the PC group (i.e., group = 1) and the C group (i.e., group = 0) is as follows:

$${ES}_{Change}=\frac{{\bar{x}}_{change.PCgroup}-{\bar{x}}_{change.Cgroup}}{S_{change.pooled}}$$

where S_change.pooled_ = $\surd \;\frac{\left(N_{1}-1\right)\times {SD}_{change.PCgroup}^{2}+\left(N_{2}-1\right)\times {SD}_{change.Cgroup}^{2}}{n_{1}+n_{2}-2}$) [56]. All analyses were performed on an intention-to-treat (ITT) data set (all participants who were randomized at T1, regardless of following or deviating from the protocol between T1–T3) and a per-protocol (PP) data set (participants who were randomized at T1 and followed the protocol between T1–T3). To compare the groups based on continuous baseline characteristics, such as age and DAS44, non-parametric Mann–Whitney U tests were performed. To compare dichotomous variables at baseline, chi-square tests were performed. The primary outcome was analyzed by means of logistic regression analysis with drug-free clinical remission at T3 (yes/no) as the outcome variable and group (PC group vs. C group) as the between-subjects predictor variable.

In order to gain insight into the effect of the intervention over time on specific time contrasts for the secondary outcomes, multilevel analyses were performed. Categorical contrasts were made for the different time points in order to compare change over time. In the first series, we tested the contrasts T0–T3 (start of the acquisition phase to the end of the tapering phase) and T0–T1 (start of the acquisition phase to the start of the conditioning phase). In the second series, we tested contrasts T1–T2 (start of conditioning phase to start of tapering phase), and T2–T3 (start of the tapering phase to the end of the tapering phase) between the two groups. In the case of values for the cytokines falling below the ELISA cut-off values, sensitivity analyses were performed. For these cytokines, a multilevel analysis was performed on the original data values and on data whereby all values below the cut-off value were set to 0. If there were no differences in significance tests, the latter analysis was reported. In the case of differences between significance tests, the results from both analyses were reported.

## 5. Conclusions

The current proof-of-principle study provided preliminary indications that pharmacological conditioning as an add-on treatment to standard treatment could optimize specific treatment outcomes, such as disease activity, in patients with recent-onset rheumatoid arthritis. Future studies could focus on conditioning designs for dosage reduction regimens with larger sample sizes, and the identification of subgroups that particularly benefit from pharmacological conditioning.

## Figures and Tables

**Table 1:** Descriptive baseline statistics per group.

	Control Group (ITT: *n* = 8; PP: *n* = 6)	Pharmacological Conditioning Group (ITT: *n* = 11 (10 for TNF-α); PP: *n* = 4)	Pairwise Comparison
**Sex, *n* female (*%*)**			
ITT	3 (37.5)	6 (54.5)	*X*^2^ = 0.54, *p* = 0.463, φ = 0.24
PP	3 (50.0)	1 (25.0)	*X*^2^ = 0.63, *p* = 0.429, φ = 0.40
**Mean (SD); Median (IQR)**			
**Age**			
ITT	57.8 (11.6); 59.1 (16.93)	61.5 (10.4); 56.7 (19.71)	*U* = 47.00, *p* = 0.840, *d* = 0.34
PP	55.6 (12.4); 58.9 (21.2)	67.8 (9.8); 68.7 (18.8)	*U* = 18.00, *p* = 0.257, *d* = 1.09
**DAS44**			
ITT	2.4 (0.7); 2.5 (1.1)	2.8 (0.6); 2.7 (0.8)	*U* = 59.50, *p* = 0.206, *d* = 0.73
PP	2.3 (0.8)2.5 (1.5)	2.8 (0.4); 2.8 (0.8)	*U* = 17.50, *p* = 0.257, *d* = 0.84
**RADAI Disease Activity**			
ITT	4.4 (1.4); 4.9 (1.9)	4.7 (1.9); 5.6 (3.6)	*U* = 57.00, *p* = 0.310, *d* = 0.18
PP	4.0 (1.5); 4.7 (2.5)	5.2 (1.9); 5.8 (3.3)	*U* = 19.00, *p* = 0.171, *d* = 0.70
**IRGL Pain**			
ITT	262.5 (32.5); 260.0 (60.0)	268.5 (60.7); 280.0 (95.0)	*U* = 49.50, *p* = 0.657, *d* = 0.12
PP	249.2 (24.6); 245.0 (46.3)	267.8 (41.6); 273.0 (79.8)	*U* = 16.00, *p* = 0.476, *d* = 0.54
**RAND36 Physical Health Composite**			
ITT	33.6 (7.7); 30.5 (14.3)	37.0 (7.0); 37.0 (13.0)	*U* = 60.00, *p* = 0.206, *d* = 0.46
PP	35.5 (8.1); 32.5 (16.0)	41.5 (4.4); 41.0 (8.5)	*U* = 18.00, *p* = 0.257, *d* = 0.93
**RAND36 Mental Health Composite**			
ITT	48.1 (8.2); 49.5 (8.5)	44.7 (10.1); 46.0 (17.0	*U* = 35.50, *p* = 0.492, *d* = −0.37
PP	48.3 (9.4); 49.5 (13.8)	47.5 (14.2); 50.5 (26.5)	*U* = 12.50, *p* = 1.000, *d* = −0.15
**CIS Fatigue Severity**			
ITT	34.4 (12.5); 36.0 (20.5)	32.9 (10.6); 34.0 (14.0)	*U* = 41.00, *p* = 0.840, *d* = −0.13
PP	34.3 (14.2); 36.0 (26.5)	26.3 (10.0); 26.5 (19.3)	*U* = 7.00, *p* = 0.352, *d* = −0.66
**IFN-γ**			
ITT	56.8 (10.0); 59.2 (18.4)	57.8 (10.1); 58.2 (18.3)	*U* = 46.00, *p* = 0.904, *d* = 0.10
PP	58.1 (9.2); 59.2 (12.3)	62.3 (12.1); 61.5 (22.6)	*U* = 14.00, *p* = 0.762, *d* = 0.39
**TNF-α**			
ITT	80.1 (10.7); 78.1 (18.1)	78.0 (12.9); 73.6 (10.4)	*U* = 35.00, *p* = 0.696, *d* = −0.18
PP	75.1 (5.5); 74.0 (9.5)	78.6 (4.8); 78.7 (9.3)	*U* = 17.00, *p* = 0.352, *d* = 0.70
**Interleukin-1β**			
ITT	2.6 (0.8); 2.3 (1.6)	2.6 (0.8); 2.38 (1.3)	*U* = 41.00, *p* = 0.840, *d* = −0.08
PP	2.5 (0.8); 2.15 (1.6)	2.7 (1.1); 2.8 (2.2)	*U* = 14.00, *p* = 0.762, *d* = 0.18
**Interleukin-6**			
ITT	23.9 (24.8); 13.5 (45.0)	33.5 (47.0); 15.76 (38.01)	*U* = 45.00, *p* = 1.000, *d* = 0.25
PP	18.9 (21.2); 10.7 (27.5)	34.5 (19.1); 41.0 (33.3)	*U* = 18.00, *p* = 0.257, *d* = 0.77

**Table 2:** Group comparisons on secondary outcome measures between T0 and T3 for the ITT and PP data sets: observed means and change scores; effect sizes of the differences between groups on the change scores. *p*-values result from the first series of the multilevel analyses.

Analyses T0–T3								
Variable	Observed Mean (SD); Median (IQR); *n*				Observed Change Score (SD; *n*)		Effect Size (SE)	Pair-Wise Comparison
	C Group		PC Group		C Group	PC Group		
	T0	T3	T0	T3	T0–T3			
**DAS44**								
ITT	2.36 (0.67); 2.50 (1.09); 8	1.27 (0.82); 1.20 (1.43); 8	2.81 (0.55); 2.70 (0.83); 11	1.65 (0.72); 1.96 (1.25); 9	1.09 (0.89; 8)	1.19 (0.71; 9)	0.12	*p* = 0.767
PP	2.31 (0.78); 2.50 (1.49); 6	1.29 (0.78); 1.19 (0.83); 6	2.83 (0.40); 2.82 (0.77); 4	1.26 (0.60); 1.00 (0.97); 4	1.02 (0.88; 6)	1.57 (0.64; 4)	0.09	*p* = 0.214
**RADAI Disease Activity**								
ITT	4.36 (1.43); 4.88 (1.93); 8	2.29 (2.07); 1.85 (2.78); 8	4.66 (1.88); 5.59 (3.55); 11	1.20 (0.68); 1.34 (1.27); 4	2.07 (1.99; 8)	3.97 (2.21; 4)	**0.92**	*p* = 0.249
PP	3.98 (1.47); 4.67 (2.51); 6	1.65 (1.35); 1.46 (2.11); 6	5.17 (1.91); 5.80 (3.31); 4	1.20 (0.68); 1.34 (1.27); 4	2.34 (1.49; 6)	3.96 (2.21; 4)	**0.91**	*p* = 0.195
**RAND Physical Health Composite**								
ITT	33.63 (7.65); 30.50 (14.25); 8	42.25 (9.33); 41.00 (17.00); 8	37.00 (6.99); 37.00 (13.00); 11	51.75 (5.74); 53.00 (10.75); 4	−8.63 (7.71; 8)	−10.25 (9.03; 4)	−0.20	*p* = 0.231
PP	35.50 (8.07); 32.50 (16.00); 6	44.50 (8.98); 44.50 (18.50); 6	41.50 (4.43); 41.00 (8.50); 4	51.75 (5.74); 53.00 (10.75); 4	−9.00 (8.10; 6)	−10.25 (9.03; 4)	−0.15	*p* = 0.764
**IRGL Pain**								
ITT	262.50 (32.51); 260.00 (60); 8	102.00 (90.19); 97.00 (129.50); 8	268.45 (60.67); 280.00 (95.00);11	83.00 (71.55); 77.50 (138.00); 4	160.50 (87.21; 8)	184.75 (90.79; 4)	0.27	*p* = 0.658
PP	249.17 (24.58); 245.00 (46.25); 6	86.00 (58.31); 97.00 (102.00); 6	267.75 (41.64); 273.00 (79.75); 4	83.00 (71.55); 77.50 (138.00); 4	163.17 (55.24; 6)	184.75 (90.88; 4)	0.31	*p* = 0.702
**CIS Fatigue Severity**								
ITT	34.38 (12.49); 36.00 (20.50); 8	23.38 (8.38); 23.00 (14.75); 8	32.91 (10.58); 34.00 (14.00); 11	18.00 (8.60); 17.50 (16.50); 4	11.00 (14.92; 8)	8.25 (12.15; 4)	−0.19	*p* = 0.762
PP	34.33 (14.19); 36.00 (26.50); 6	20.83 (8.11); 21.00 (12.75); 6	26.25 (10.01); 26.50 (19.25); 4	18.00 (8.60); 17.50 (16.50); 4	13.50 (16.54; 6)	8.25 (12.14; 4)	−0.35	*p* = 0.612
**RAND Mental Health Composite**								
ITT	48.13 (8.18); 49.50 (8.50); 8	51.88 (6.51); 54.50 (9.75); 8	44.73 (10.11); 46.00 (17.00); 11	55.25 (4.11); 55.50 (7.75); 4	−3.75 (10.40; 8)	−7.75 (10.44; 4)	−0.38	*p* = 0.197
PP	48.33 (9.42); 49.50 (13.75); 6	53.50 (4.59); 54.50 (9.50); 6	47.50 (14.20); 50.50 (26.50); 4	55.25 (4.11); 55.50 (7.75); 4	−5.17 (9.43; 6)	−7.75 (10.44; 4)	−0.26	*p* = 0.635
**IFN-γ**								
ITT	56.76 (9.99); 59.25 (18.37); 8	47.10 (3.62); 46.23 (4.82); 8	57.79 (10.08); 58.16 (18.29); 11	50.32 (7.01); 48.98 (3.65); 9	9.67 (9.48; 8)	5.75 (9.49; 9)	−0.41	*p* = 0.719
PP	58.05 (9.23); 59.25 (12.32); 6	47.39 (4.19); 46.65 (6.59); 6	62.25 (12.06); 61.49 (22.58); 4	50.17 (1.39); 49.82 (NA); 3	10.66 (9.06; 6)	9.69 (12.19; 3)	−0.10	*p* = 0.866
**TNF-α**								
ITT	80.12 (10.68); 78.08 (18.07); 8	211.57 (223.92); 97.61 (305.54); 8	78.01 (12.94); 73.58 (10.40); 10	91.20 (7.00); 93.68 (12.25); 8	−131.45 (227.73; 8)	−12.63 (13.47; 8)	**0.74**	***p*** **= 0.030**
PP	75.11 (5.52); 74.00 (9.52); 6	251.91 (249.76); 104.48 (442.27); 6	78.63 (4.78); 78.68 (9.27); 4	95.63 (2.52); 94.81 (NA); 3	−176.80 (250.42; 6)	−17.59 (3.19; 3)	**0.75**	*p* = 0.123
**Interleukin-1β**								
ITT	2.65 (0.80); 2.29 (1.56); 8	2.45 (0.30); 2.34 (0.52); 8	2.59 (0.79); 2.38 (1.34); 11	2.72 (0.70); 2.55 (0.51); 8	0.20 (0.95; 8)	−0.02 (0.89; 8)	−0.24	*p* = 0.376
PP	2.53 (0.82); 2.15 (1.61); 6	2.38 (0.25); 2.30 (0.31); 6	2.71 (1.13); 2.83 (2.17); 4	2.48 (0.12); 2.53 (NA); 3	0.15 (0.95; 6)	0.34 (1.24; 3)	0.18	*p* = 0.889
**Interleukin-6**								
ITT	23.92 (24.82); 13.48 (44.97); 8	9.30 (6.36); 7.33 (4.63); 8	33.47 (47.00); 15.76 (38.01); 11	15.57 (16.19); 9.53 (8.29); 8	14.61 (27.28; 8)	23.96 (56.64; 8)	0.21	*p* = 0.824
PP	18.91 (21.16); 10.70 (27.51); 6	7.52 (1.89); 7.33 (3.12); 6	34.46 (19.06); 41.00 (33.32); 4	22.84 (27.60); 7.69 (NA); 3	11.39 (21.19; 6)	10.42 (28.65; 3)	−0.04	*p* = 0.993

Note: NA = IQR not calculated due to insufficient observations.

**Table 3:** Group comparisons on secondary outcome measures between T0 and T1 for the ITT and PP data sets: observed means and change scores; effect sizes of the differences between groups on the change scores; and results of the first series of the multilevel analyses. *p*-values result from the first series of the multilevel analyses.

**Analyses T0–T1**								
**Variable**	**Observed Mean (SD); Median (IQR); *n***				**Observed Change Score (SD; *n*)**		**Effect Size (SE)**	**Pair-Wise Comparison**
	**C Group**		**PC Group**		**C Group**	**PC Group**		
	**T0**	**T1**	**T0**	**T1**	**T0–T1**			
**DAS44**								
ITT	2.36 (0.67); 2.50 (1.09); 8	0.99 (0.38); 0.89 (0.66); 8	2.81 (0.55); 2.70 (0.83); 11	0.92 (0.49); 0.86 (0.61); 11	1.37 (0.61; 8)	1.88 (0.40; 11)	**1.04**	*p* = 0.083
PP	2.31 (0.78); 2.50 (1.49); 6	0.95 (0.38); 0.89 (0.70); 6	2.83 (0.40); 2.82 (0.77); 4	0.70 (0.48); 0.62 (0.89); 4	1.36 (0.68; 6)	2.13 (0.36; 4)	**1.32**	*p* = 0.085
**RADAI Disease Activity**								
ITT	4.36 (1.43); 4.88 (1.93); 8	2.19 (1.52); 1.92 (1.97); 8	4.66 (1.88); 5.59 (3.55); 11	2.22 (2.09); 1.31 (3.70); 9	2.10 (1.96; 8)	3.16 (2.53; 4)	**0.50**	*p* = 0.724
PP	3.98 (1.47); 4.67 (2.51); 6	1.98 (1.74); 1.41 (2.03); 6	5.17 (1.91); 5.80 (3.31); 4	2.04 (2.30); 1.60 (NA); 3	2.01 (1.78; 6)	4.05 (1.89; 3)	**1.13**	*p* = 0.323
**RAND Physical Health Composite**								
ITT	33.63 (7.65); 30.50 (14.25); 8	39.13 (5.17); 41.50 (9.00); 8	37.00 (6.99); 37.00 (13.00); 11	41.44 (2.51); 42.00 (2.50); 9	−5.50 (5.32; 8)	−6.89 (5.84; 9)	−0.25	*p* = 0.987
PP	35.50 (8.07); 32.50 (16.00); 6	41.50 (2.95); 42.00 (4.25); 6	41.50 (4.43); 41.00 (8.50); 4	41.00 (1.73); 42.00 (NA); 3	−6.00 (6.07; 6)	−1.33 (4.73; 3)	**0.82**	*p* = 0.180
**IRGL Pain**								
ITT	262.50 (32.51); 260.00 (60); 8	87.25 (93.83); 62.50 (112.25); 8	268.45 (60.67); 280.00 (95.00); 11	70.56 (77.92); 50.00 (147.50); 9	175.25 (79.67; 8)	203.67 (120.33; 9)	0.27	*p* = 0.609
PP	249.17 (24.58); 245.00 (46.25); 6	76.83 (107.25); 36.50 (110.00); 6	267.75 (41.64); 273.00 (79.75); 4	61.33 (95.84); 6.00 (NA); 3	172.33 (93.19; 6)	224.00 (95.69; 3)	**0.55**	*p* = 0.541
**CIS Fatigue Severity**								
ITT	34.38 (12.49); 36.00 (20.50); 8	28.97 (15.34);28.33 (27.25); 8	32.91 (10.58); 34.00 (14.00); 11	20.98 (9.92); 22.86 (18.00); 9	5.41 (18.41; 8)	15.13 (14.38; 9)	**0.59**	*p* = 0.336
PP	34.33 (14.19); 36.00 (26.50); 6	30.45 (17.22);28.33 (33.59); 6	26.25 (10.01); 26.50 (19.25); 4	17.33 (8.08); 16.00 (NA); 4	3.88 (21.51; 6)	13.00 (13.23; 3)	0.47	*p* = 0.566
**RAND Mental Health Composite**								
ITT	48.13 (8.18); 49.50 (8.50); 8	39.38 (4.57); 39.00 (6.00); 8	44.73 (10.11); 46.00 (17.00); 11	38.33 (3.54); 39.00 (3.50); 9	8.75 (7.61; 8)	3.56 (10.09; 9)	**−0.58**	*p* = 0.461
PP	48.33 (9.42); 49.50 (13.75); 6	41.17 (3.19);40.00 (5.75); 6	47.50 (14.20); 50.50 (26.50); 4	39.00 (1.73); 40.00 (NA); 3	7.17 (7.22; 6)	4.00 (14.73; 3)	−0.32	*p* = 0.972
**IFN-γ**								
ITT	56.76 (9.99); 59.25 (18.37); 8	60.55 (13.95); 68.46 (27.07); 8	57.79 (10.08); 58.16 (18.29); 11	64.06 (13.07); 68.54 (24.78); 11	−3.78 (19.18; 8)	−6.27 (15.83; 11)	−0.14	*p* = 0.670
PP	58.05 (9.23);59.25 (12.32); 6	57.60 (15.19); 55.99 (28.59); 6	62.25 (12.06); 61.49 (22.58); 4	72.56 (5.29); 70.71 (9.21); 4	−0.46 (19.58; 6)	−10.31 (9.91; 4)	**−0.65**	*p* = 0.187
**TNF-α**								
ITT	80.12 (10.68); 78.08 (18.07); 8	78.08 (5.32); 77.81 (9.02); 8	78.01 (12.94); 73.58 (10.40); 10	74.95 (6.72); 74.96 (13.46); 10	2.04 (14.61; 8)	3.07 (13.74; 10)	0.07	*p* = 0.984
PP	75.11 (5.52); 74.00 (9.52); 6	79.81 (4.50); 80.37 (9.29); 6	78.63 (4.78); 78.68 (9.27); 4	73.33 (8.65); 70.82 (15.76); 4	−4.70 (8.95; 6)	5.30 (6.08; 4)	**1.25**	*p* = 0.917
**Interleukin-1β**								
ITT	2.65 (0.80); 2.29 (1.56); 8	2.19 (0.24); 2.29 (0.35); 8	2.59 (0.79); 2.38 (1.34); 11	2.23 (0.33); 2.17 (0.58); 11	0.45 (0.84; 8)	0.35 (1.04; 11)	−0.10	*p* = 0.781
PP	2.53 (0.82); 2.15 (1.61); 6	2.12 (0.24); 2.20 (0.40); 6	2.71 (1.13); 2.83 (2.17); 4	2.04 (0.24); 2.06 (0.20); 4	0.41 (1.05; 6)	0.67 (1.31; 4)	0.22	*p* = 0.604
**Interleukin-6**								
ITT	23.92 (24.82); 13.48 (44.97); 8	7.54 (3.19); 6.32 (5.50); 8	33.47 (47.00); 15.76 (38.01); 11	6.67 (2.38); 5.94 (0.72); 11	16.38 (23.49; 8)	26.81 (46.33; 11)	0.27	*p* = 0.466
PP	18.91 (21.16); 10.70 (27.51); 6	5.90 (1.09); 5.91 (1.83); 6	34.46 (19.06); 41.00 (33.32); 4	6.15 (0.23); 6.14 (0.41); 4	13.02 (20.42; 6)	28.32 (18.92; 4)	**0.77**	*p* = 0.189

Note: NA = IQR not calculated due to insufficient observations.

**Table 4:** Group comparisons on secondary outcome measures between T1 and T2 for the ITT and PP data sets: observed means and change scores; effect sizes of the differences between groups on the change scores; and results of the second series of the multilevel analyses. *p*-values result from the second series of the multilevel analyses.

Analyses T1–T2								
Variable	Observed Mean (SD); Median (IQR); *n*				Observed Change Score (SD)		Effect Size (SE)	Pair-Wise Comparison
	C Group		PC Group		C Group	PC Group		
	T1	T2	T1	T2	T1–T2			
**DAS44**								
ITT	0.99 (0.38); 0.89 (0.66); 8	0.86 (0.57); 0.65 (0.82); 8	0.92 (0.49); 0.86 (0.61); 11	1.04 (0.70); 0.90 (1.01); 10	0.14 (0.53; 8)	−0.10 (0.53; 10)	−0.43	*p* = 0.427
PP	0.95 (0.38); 0.89 (0.70); 6	0.96 (0.62); 0.77 (0.99); 6	0.70 (0.48); 0.62 (0.89); 4	0.45 (0.17); 0.48 (0.32); 4	−0.01 (0.52; 6)	0.26 (0.63; 4)	0.47	*p* = 0.539
**RADAI Disease Activity**								
ITT	2.19 (1.52); 1.92 (1.97); 8	2.26 (1.36); 2.11 (2.10); 8	2.22 (2.09); 1.31 (3.70); 9	1.98 (1.75); 1.62 (3.29); 4	−0.07 (1.60; 8)	−0.31 (1.09; 4)	−0.16	*p* = 0.881
PP	1.98 (1.74); 1.41 (2.03); 6	2.37 (1.58); 2.66 (2.64); 6	2.04 (2.30); 1.60 (NA); 3	1.86 (2.12); 0.88 (0.48); 3	−0.39 (1.75; 6)	0.19 (0.56; 3)	0.38	*p* = 0.671
**RAND Physical Health Composite**								
ITT	39.13 (5.17); 41.50 (9.00); 8	39.38 (6.00); 42.00 (11.75); 8	41.44 (2.51); 42.00 (2.50); 9	40.00 (3.46); 41.00 (6.00); 4	−0.25 (2.43; 8)	1.25 (4.03; 4)	**0.50**	*p* = 0.360
PP	41.50 (2.95); 42.00 (4.25); 6	41.83 (4.58); 43.50 (6.00); 6	41.00 (1.73); 42.00 (NA); 3	41.67 (1.15); 41.00 (NA); 3	−0.33 (2.16; 6)	−0.67 (1.53; 3)	−0.17	*p* = 0.942
**IRGL Pain**								
ITT	87.25 (93.83); 62.50 (112.25); 8	112.75 (84.98); 106.50 (142.75); 8	70.56 (77.92); 50.00 (147.50); 9	96.50 (81.76); 65.50 (141.00); 4	−25.50 (114.46; 8)	−37.00 (7.79; 4)	−0.12	*p* = 0.898
PP	76.83 (107.25); 36.50 (110.00); 6	109.00 (89.59); 106.50 (143.75); 6	61.33 (95.84); 6.00 (NA); 3	101.33 (99.43); 49.00 (NA); 3	−32.17 (119.36; 6)	−40.00 (6.08; 3)	0.08	*p* = 0.899
**CIS Fatigue Severity**								
ITT	28.97 (15.34); 28.33 (27.25); 8	24.63 (14.39); 17.00 (24.75); 8	20.98 (9.92); 22.86 (18.00); 9	20.00 (6.27); 19.50 (12.00); 4	4.34 (11.70; 8)	1.00 (6.06; 4)	−0.32	*p* = 0.651
PP	30.45 (17.22); 28.33 (33.59); 6	24.17 (15.78); 16.50 (29.00); 6	17.33 (8.08); 16.00 (NA); 4	17.33 (4.04); 18.00 (NA); 3	6.29 (7.13; 6)	0.00 (7.00; 3)	**−0.89**	*p* = 0.579
**RAND Mental Health Composite**								
ITT	39.38 (4.57); 39.00 (6.00); 8	37.75 (2.55); 37.50 (3.75); 8	38.33 (3.54); 39.00 (3.50); 9	36.75 (4.03); 37.00 (7.75); 4	1.63 (5.50; 8)	0.50 (3.11; 4)	−0.23	*p* = 0.993
PP	41.17 (3.19); 40.00 (5.75); 6	37.83 (2.86); 37.50 (5.00); 6	39.00 (1.73); 40.00 (NA); 3	38.33 (3.06); 39.00 (NA); 3	3.33 (5.24; 6)	0.67 (3.79; 3)	**−0.55**	*p* = 0.655
**IFN-γ**								
ITT	60.55 (13.95); 68.46 (27.07); 8	45.93 (2.46); 46.94 (4.01); 7)	64.06 (13.07); 68.54 (24.78); 11	48.03 (8.93); 45.16 (5.85); 10	17.00 (14.55; 7)	14.41 (14.18; 10)	−0.18	*p* = 0.921
PP	57.60 (15.19); 55.99 (28.59); 6	46.07 (1.88); 46.94 (3.61); 5	72.56 (5.29); 70.71 (9.21); 4	43.85 (0.94); 43.74 (NA); 3	14.28 (16.71; 5)	26.14 (1.46; 3)	**0.87**	*p* = 0.053
**TNF-α**								
ITT	78.08 (5.32); 77.81 (9.02); 8	89.21 (13.69); 84.61 (27.84); 7	74.95 (6.72); 74.96 (13.46); 10	89.20 (14.93); 83.18 (11.92); 9	−11.63 (11.65; 7)	−15.38 (14.75; 9)	−0.28	*p* = 0.954
PP	79.81 (4.50); 80.37 (9.29); 6	88.26 (13.43); 84.61 (21.26); 5	73.33 (8.65); 70.82 (15.76); 4	95.89 (26.51); 83.18 (NA); 3	−8.80 (10.71; 5)	−26.50 (22.13; 3)	**−1.14**	*p* = 0.894
**Interleukin-1β**								
ITT	2.19 (0.24); 2.29 (0.35); 8	2.74 (0.51); 2.52 (0.68); 7	2.23 (0.33); 2.17 (0.58); 11	2.56 (0.38); 2.54 (0.43); 9	−0.56 (0.60; 7)	−0.39 (0.36; 9)	0.36	*p* = 0.579
PP	2.12 (0.24); 2.20 (0.40); 6	2.62 (0.34); 2.52 (0.67); 5	2.04 (0.24); 2.06 (0.20); 4	2.47 (0.21); 2.54 (NA); 3	−0.54 (0.56; 5)	−0.52 (0.34; 3)	0.03	*p* = 0.899
**Interleukin-6**								
ITT	7.54 (3.19); 6.32 (5.50); 8	6.07 (1.07); 5.90 (1.34); 7	6.67 (2.38); 5.94 (0.72); 11	8.69 (6.42); 6.29 (3.52); 9	1.70 (3.45; 7)	−1.67 (4.43, 9)	**−0.83**	*p* = 0.814
PP	5.90 (1.09); 5.91 (1.83); 6	6.02 (2.12); 4.89 (3.01); 5	6.15 (0.23); 6.14 (0.41); 4	5.61 (0.61); 5.43 (NA); 3	−0.13 (1.79; 5)	0.60 (0.82; 3)	0.48	*p* = 0.887

Note: NA = IQR not calculated due to insufficient observations.

**Table 5:** Group comparisons on secondary outcome measures between T2 and T3 for the ITT and PP data sets: observed means and change scores; effect sizes of the differences between groups on the change scores; and results of the second series of the multilevel analyses. *p*-values result from the second series of the multilevel analyses.

Analyses T2–T3								
Variable	Observed Mean (SD); Median (IQR); *n*				Observed Change Score (SD)		Effect Size (SE)	Pair-Wise Comparison
	C Group		PC Group		C Group	PC Group		
	T2	T3	T2	T3	T2–T3			
**DAS44**								
ITT	0.86 (0.57); 0.65 (0.82); 8	1.27 (0.82); 1.20 (1.43); 8	1.04 (0.70); 0.90 (1.01); 10	1.65 (0.72); 1.96 (1.25); 9	−0.41 (0.54; 8)	−0.61 (0.52; 9)	−0.38	*p* = 0.533
PP	0.96 (0.62); 0.77 (0.99); 6	1.29 (0.78); 1.19 (0.83); 6)	0.45 (0.17); 0.48 (0.32); 4	1.26 (0.60); 1.00 (0.97); 4	−0.32 (0.40; 6)	−0.81 (0.54; 4)	**−1.06**	*p* = 0.264
**RADAI Disease Activity**								
ITT	2.26 (1.36); 2.11 (2.10); 8	2.29 (2.07); 1.85 (2.78); 8	1.98 (1.75); 1.62 (3.29); 4	1.20 (0.68); 1.34 (1.27); 4	−0.03 (2.49; 8)	0.81 (1.46; 3)	0.37	*p* = 0.529
PP	2.37 (1.58); 2.66 (2.64); 6	1.65 (1.35); 1.46 (2.11); 6	1.86 (2.12); 0.88 (0.48); 3	1.20 (0.68); 1.34 (1.27); 4	0.72 (2.12; 6)	0.81 (1.46; 3)	0.05	*p* = 0.846
**RAND Physical Health Composite**								
ITT	39.38 (6.00); 42.00 (11.75); 8	42.25 (9.33); 41.00 (17.00); 8	40.00 (3.46); 41.00 (6.00); 4	51.75 (5.74); 53.00 (10.75); 4	−2.88 (6.44; 8)	−10.33 (6.66; 3)	**−1.15**	*p* = 0.061
PP	41.83 (4.58); 43.50 (6.00); 6	44.50 (8.98); 44.50 (18.50); 6	41.67 (1.15); 41.00 (NA); 3	51.75 (5.74); 53.00 (10.75); 4	−2.67 (6.77; 6)	−10.33 (6.66; 3)	**−1.14**	*p* = 0.125
**IRGL Pain**								
ITT	112.75 (84.98); 106.50 (142.75); 8	102.00 (90.19); 97.00 (129.50); 8	96.50 (81.76); 65.50 (141.00); 4	83.00 (71.55); 77.50 (138.00); 4	10.75 (80.20; 8)	26.00 (23.30; 3)	0.21	*p* = 0.905
PP	109.00 (89.59); 106.50 (143.75); 6	86.00 (58.31); 97.00 (102.00); 6	101.33 (99.43); 49.00 (NA); 3	83.00 (71.55); 77.50 (138.00); 4	23.00 (83.03; 6)	26.00 (23.30; 3)	0.04	*p* = 0.903
**CIS Fatigue Severity**								
ITT	24.63 (14.39); 17.00 (24.75); 8	23.38 (8.38); 23.00 (14.75); 8	20.00 (6.27); 19.50 (12.00); 4	18.00 (8.60); 17.50 (16.50); 4	1.25 (17.59; 8)	−3.67 (10.26; 3)	−0.30	*p* = 0.948
PP	24.17 (15.78); 16.50 (29.00); 6	20.83 (8.11); 21.00 (12.75); 6	17.33 (4.04); 18.00 (NA); 3	18.00 (8.60); 17.50 (16.50); 4	3.33 (18.83; 6)	−3.67 (10.26; 3)	−0.42	*p* = 0.631
**RAND Mental Health Composite**								
ITT	37.75 (2.55); 37.50 (3.75); 8	51.88 (6.51); 54.50 (9.75); 8	36.75 (4.03); 37.00 (7.75); 4	55.25 (4.11); 55.50 (7.75); 4	−14.13 (5.79; 8)	−15.33 (0.58; 3)	−0.24	*p* = 0.547
PP	37.83 (2.86); 37.50 (5.00); 6	53.50 (4.59); 54.50 (9.50); 6	38.33 (3.06); 39.00 (NA); 3	55.25 (4.11); 55.50 (7.75); 4	−15.67 (4.32; 6)	−15.33 (0.58; 3)	0.09	*p* = 0.983
**IFN-γ**								
ITT	45.93 (2.46); 46.94 (4.01); 7	47.10 (3.62); 46.23 (4.82); 8	48.03 (8.93); 45.16 (5.85); 10	50.32 (7.01); 48.98 (3.65); 9	−1.72 (4.45; 7)	−1.81 (4.55; 8)	−0.02	*p* = 0.965
PP	46.07 (1.88); 46.94 (3.61); 5	47.39 (4.19); 46.65 (6.59); 6	43.85 (0.94); 43.74 (NA); 3	50.17 (1.39); 49.82 (0.84); 3	−2.15 (4.48; 5)	−5.11 (1.37; 2)	**−0.73**	*p* = 0.577
**TNF-α**								
ITT	89.21 (13.69); 84.61 (27.84); 7	211.57 (223.92); 97.61 (305.54); 8	89.20 (14.93); 83.18 (11.92); 9	91.20 (7.00); 93.68 (12.25); 8	−59.99 (149.96; 7)	−0.09 (17.76; 7)	**0.56**	***p*** **= 0.032**
PP	88.26 (13.43); 84.61 (21.26); 5	251.91 (249.76); 104.48 (442.27); 6	95.89 (26.51); 83.18 (NA); 3	95.63 (2.52); 94.81 (1.19); 3	−84.40 (176.04; 5)	8.04 (33.27; 2)	**0.58**	*p* = 0.135
**Interleukin-1β**								
ITT	2.74 (0.51); 2.52 (0.68); 7	2.45 (0.30); 2.34 (0.52); 8	2.56 (0.38); 2.54 (0.43); 9	2.72 (0.70); 2.55 (0.51); 8	0.25 (0.50; 7)	−0.28 (0.84; 7)	**−0.77**	*p* = 0.259
PP	2.62 (0.34); 2.52 (0.67); 5	2.38 (0.25); 2.30 (0.31); 6	2.47 (0.21); 2.54 (NA); 3	2.48 (0.12); 2.53 (0.03); 3	0.20 (0.55; 5)	0.04 (0.04; 2)	−0.33	*p* = 0.648
**Interleukin-6**								
ITT	6.07 (1.07); 5.90 (1.34); 7	9.30 (6.36); 7.33 (4.63); 8	8.69 (6.42); 6.29 (3.52); 9	15.57 (16.19); 9.53 (8.29); 8	−3.64 (6.94; 7)	−7.66 (19.44; 7)	−0.27	*p* = 0.813
PP	6.02 (2.12); 4.89 (3.01); 5	7.52 (1.89); 7.33 (3.12); 6	5.61 (0.61); 5.43 (NA); 3	22.84 (27.60); 7.69 (1.55); 3	−1.72 (3.01; 5)	−24.56 (33.73; 2)	**−1.49**	*p* = 0.195

Note: NA = IQR not calculated due to insufficient observations.

## Data Availability

The data that support the findings of this study are available upon request from the corresponding author, A.W.M.E. The data are not publicly available due to their containing information that could compromise the privacy of research participants. Program codes used for analyses are also available on request from the corresponding author, A.W.M.E. Data requests will be evaluated per case and in collaboration with privacy and legal experts.
